# Prognostic Value of Circulating Tumor DNA in HR+/HER2− Stage I–III Breast Cancer: A Systematic Review

**DOI:** 10.3390/cancers17172831

**Published:** 2025-08-29

**Authors:** Ismail Ajjawi, Mariya Rozenblit, Alejandro Rios-Hoyo, Maryam B. Lustberg

**Affiliations:** 1 Yale School of Medicine, Yale University, New Haven, CT 06510, USA; ismail.ajjawi@yale.edu; 2 Yale Cancer Center, Yale School of Medicine, New Haven, CT 06510, USA; mariya.rozenblit@yale.edu (M.R.); alriho@gmail.com (A.R.-H.); 3 Department of Medicine and Life Sciences, Universitat Pompeu Fabra (UPF), 08003 Barcelona, Spain

**Keywords:** circulating tumor DNA, early-stage breast cancer, endocrine therapy, recurrence risk, liquid biopsy

## Abstract

**Background**: Hormone receptor-positive (HR+), HER2-negative breast cancer accounts for the majority of breast cancer diagnoses. While outcomes have improved with neoadjuvant and adjuvant therapies, the risk of late recurrence persists, and there remains a critical need for reliable biomarkers to guide prognosis and post-treatment surveillance. Circulating tumor DNA (ctDNA), detectable via liquid biopsy, has emerged as a promising tool for monitoring minimal residual disease and predicting survival outcomes. This systematic review evaluates the association between ctDNA detection during neoadjuvant or adjuvant treatment and survival outcomes in early-stage HR+/HER2− breast cancer. **Methods**: This systematic review was conducted in accordance with PRISMA guidelines. A comprehensive literature search of Ovid MEDLINE and Embase was conducted to identify studies published through 3 May 2024 that evaluated ctDNA as a prognostic biomarker in stage I–III HR+/HER2− breast cancer. We included studies reporting recurrence-free survival, invasive disease-free survival, or overall survival and excluded non-original studies, conference abstracts, and non-English articles. Data extraction and qualitative synthesis were performed, and the risk of bias was qualitatively assessed across studies. No review protocol was registered. **Results**: Eleven studies comprising 1644 patients met the inclusion criteria. In the neoadjuvant setting, ctDNA positivity prior to treatment initiation was associated with inferior survival outcomes. In the adjuvant setting, detection of ctDNA during or after treatment was consistently linked to poorer recurrence-free and invasive disease-free survival. Across studies, ctDNA detection was a significant negative prognostic marker. **Conclusions**: This systematic review supports the prognostic value of ctDNA in HR+/HER2− early-stage breast cancer. Limitations include small sample sizes, observational study designs, and heterogeneity in ctDNA assays. Standardization of ctDNA testing methods and further prospective trials are needed to validate its clinical utility and explore its potential role in guiding therapeutic interventions.

## Simple Summary

Hormone receptor-positive, HER2 (human epidermal growth factor receptor 2)-negative breast cancer represents the most common subtype of breast cancer. While outcomes have improved with advances in systemic therapies, late recurrences—defined as disease recurrence occurring more than five years after initial diagnosis—remain a significant clinical concern. Circulating tumor DNA (ctDNA), detectable through a blood test, has emerged as a promising biomarker for monitoring minimal residual disease and predicting recurrence. This systematic review evaluates whether ctDNA detection during or after treatment is associated with survival outcomes in patients with early-stage (I–III) hormone receptor-positive, HER2-negative breast cancer. The findings suggest that ctDNA positivity correlates with poorer recurrence-free and overall survival, highlighting its potential utility as a prognostic tool. However, further research is needed to determine its role in guiding treatment decisions and routine clinical use.

## 1. Introduction

Breast cancer remains one of the most prevalent and challenging malignancies, being the most frequently diagnosed cancer among females and the second leading cause of cancer-related death, following lung cancer [1]. Although mortality has decreased by over 40% in the past two decades and screening has reduced mortality by an estimated 25%, the overall burden remains significant. Additionally, a noticeable increase in incidence has been reported in women younger than 50 years [2,3]. Projections indicate that by 2050, new cases and deaths in the United States are expected to increase by 38% and 68%, respectively [4]. Among breast cancer subtypes, hormone receptor-positive (HR+), HER2-negative tumors comprise more than two-thirds of cases. In this HR+/HER2-negative population, approximately 50% of cases are diagnosed at stage I, 35% at stage II, 12% at stage III, and 3% at stage IV, reflecting that most patients are diagnosed during routine screening [5].

As treatments for high-risk breast cancer (stage IIB and stage III) have improved with novel adjuvant therapies, recurrences from these stages have decreased. However, metastatic recurrence remains a significant concern, particularly from stage I and stage II breast cancer [5], with a notably elevated risk of late recurrence in hormone-positive breast cancer, where the risk persists for years [6]. As cure rates for early-stage breast cancer are high and adjuvant treatments have improved for stage IIB and stage III breast cancer, since 2003, most cancer-related deaths have been attributed to metastatic recurrence originating from stages I and II [5].

Systemic therapies administered both in the neoadjuvant and adjuvant settings have significantly improved outcomes for patients with HR+/HER2− breast cancer. Neoadjuvant therapy is administered in locally advanced breast cancers prior to surgery with the goals of reducing tumor size and assessing treatment response. This treatment is used across all subtypes of breast cancer; however, it is more frequently used in triple-negative and HER2+ compared to HR+/HER2− breast cancers.

In HR+/HER2− breast cancers, it typically involves sequential treatment with chemotherapy (combining taxanes with or without anthracyclines) followed by endocrine therapy [7,8,9,10,11,12,13,14,15,16,17,18,19,20,21,22]. This strategy not only facilitates breast-conserving surgery but also provides early insights into the effect of these therapies on the tumor. Pathologic complete response (pCR) is an established prognostic marker in TNBC and HER2+ subtypes, strongly correlating with long-term outcomes. In contrast, HR+/HER2− tumors generally achieve pCR at much lower rates. Although patients with HR+/HER2− tumors who do achieve pCR often exhibit excellent long-term outcomes, failure to achieve pCR does not reliably predict poor prognosis in this subtype [23,24].

In HR+/HER2− cases that received neoadjuvant treatment, adjuvant therapy (given after surgery) includes endocrine treatments such as tamoxifen, aromatase inhibitors, and/or ovarian suppression in premenopausal patients [25,26]. Different genomic assays have been designed to guide decisions for adjuvant endocrine and chemotherapy in patients with ER+HER2− breast cancer, such as Oncotype DX (RS) and Mamma Print (MP), among others. However, these assays were not designed to predict the risk of late recurrence beyond 5–10 years post-diagnosis. This limitation stems from the fact that the original validation cohorts primarily captured early events, their predictive algorithms focus on early recurrence rather than late recurrence, and late recurrences may involve biologically distinct, dormant tumor cells that are not detected by these tests. Therefore, their utility in long-term risk stratification is limited [27]. In addition, the adjuvant use of CDK (cyclin-dependent kinase) 4/6 inhibitors—such as ribociclib or abemaciclib—has notably improved event-free survival in high-risk HR+/HER2− breast cancer [11,12,13,14,15,16]. Following treatment, the current National Comprehensive Cancer Network (NCCN) Breast Cancer Guidelines Version 3.2024 and European Society of Medical Oncology (ESMO) 2023 Clinical Practice Guidelines recommend annual bilateral or contralateral mammography in addition to ultrasound or magnetic resonance imaging when indicated; however, in asymptomatic patients, other imaging studies are not recommended [28,29].

While these treatments have advanced the care of HR+/HER2− breast cancer, there remains a need for reliable predictors of treatment response and long-term prognosis to better tailor patient management. Furthermore, late recurrences, some occurring decades after initial diagnosis [6], pose a significant unmet clinical need.

Circulating tumor DNA (ctDNA) has emerged as a promising biomarker in cancer research and clinical practice. This non-invasive tool provides real-time insights into tumor genetics through small fragments of DNA released into the bloodstream from cancer cells [30,31,32,33,34,35,36]. The ability to monitor tumor dynamics, treatment responses, clonal heterogeneity, and resistance mechanisms represents a promising potential for further enhancing our understanding of the mechanisms of breast tumor minimal residual disease [37,38,39,40,41,42,43,44,45,46].

The analysis of ctDNA could potentially allow clinicians to better understand tumor heterogeneity, monitor disease relapse or progression, and potentially predict treatment response or resistance. This evolving field of ctDNA analysis is predicted to significantly advance personalized cancer therapy, offering more precise interventions tailored to individual patients and improving overall cancer care outcomes, if further validated in well-designed interventional trials.

Different studies have evaluated the prognostic effect of ctDNA detection on localized breast cancer, including different subtypes of breast cancer (e.g., HR+/HER2+ breast cancer, triple negative breast cancer, among others). However, few studies have explored the relationship between ctDNA presence, its dynamics during treatment, and survival outcomes in early-stage (I–III) HR+/HER2− breast cancer patients.

The primary objective of this systematic review is to determine whether the detection of ctDNA in blood liquid biopsy during the neoadjuvant or adjuvant treatment setting is associated with survival outcomes (including recurrence-free survival and overall survival) in patients with HR+/HER2− stages I–III breast cancer. By systematically evaluating existing studies, this review aims to provide a clearer understanding of how ctDNA detection correlates with survival metrics.

## 2. Materials and Methods

### 2.1. Eligibility Criteria

This systematic review considered studies involving patients with HR+/HER2-negative breast cancer stages I–III from 2000 to 3 May 2024. Eligible studies focused on neoadjuvant or adjuvant chemotherapy, endocrine therapy, and CDK4/6 inhibitors and included measurements of circulating tumor DNA (ctDNA). Studies had to specifically report on recurrence/disease/invasive disease-free survival and overall survival. Only peer-reviewed articles—randomized controlled trials, cohort studies, and case–control studies and abstracts published in English—were included. Only peer-reviewed conference abstracts published in official journal proceedings of scientific meetings (e.g., ASCO, SABCS) were included. Non-peer-reviewed abstracts, preprints, theses, dissertations, clinical trial registries, and unpublished reports were excluded. Studies including multiple breast cancer subtypes were only included if results for HR+/HER2− early-stage breast cancer were reported separately. Studies without stratified results were excluded to ensure the review focused solely on this patient population. Studies were excluded if they addressed non-HR+/HER2− subtypes or different stages of breast cancer, did not measure ctDNA, did not report relevant survival outcomes, or were review articles.

### 2.2. Information Sources

To identify relevant studies for this systematic review, a comprehensive search was conducted across multiple information sources. Two primary databases were utilized: Ovid MEDLINE and Embase. These databases were chosen because together they provide comprehensive coverage of biomedical and oncology literature and overlap substantially with other indexing services such as Web of Science and Scopus. The search in Ovid MEDLINE and Embase covered the period from 2000 to 3 May 2024. In addition to these databases, reference lists of relevant articles and review papers were examined to identify additional studies that might not have been captured through the database searches. All sources were last searched or consulted on 3 May 2024, ensuring that the search was up-to-date and inclusive of the most recent research.

### 2.3. Search Strategy

The search strategies for Ovid MEDLINE and Embase were designed to capture studies related to circulating tumor DNA (ctDNA), neoadjuvant and adjuvant chemotherapy, and HR+/HER2− breast cancer. For Ovid MEDLINE, the search terms included “circulating tumor DNA” or variations like “ctdna” and “cfdna,” combined with terms for chemotherapy and breast cancer subtypes, using the following search strategy: (1) circulating tumor dna/or (ctdna or cfdna or (Circulating adj3 DNA)); (2) chemotherapy, adjuvant/or neoadjuvant therapy/or (Neoadjuvant or adjuvant); (3) exp Breast neoplasms/or Triple negative breast neoplasms/or (Breast adj3 (cancer* or neoplas* or tumor* or tumour*)); (4) (hormone receptor positive or her2 negative or “HR-positive/HER2-negative” or “HR+/HER2-negative”); and combined as (5) 3 or 4 and (6) 1 and 2 and 5. For Embase, the search terms included similar concepts with additional terms for adjuvant and neoadjuvant therapies using the following strategy: (1) circulating tumor dna/or (ctdna or cfdna or (Circulating adj3 DNA)); (2) adjuvant chemotherapy/or cancer adjuvant chemotherapy/or neoadjuvant therapy/or (neoadjuvant or adjuvant); (3) exp breast tumor/or triple negative breast cancer/or (Breast adj3 (cancer* or neoplas* or tumor* or tumour*)); (4) (hormone receptor positive or her2 negative or “HR-positive/HER2-negative” or “HR+/HER2-negative”); and combined as (5) 3 or 4 and (6) 1 and 2 and 5. Filters and limits used included peer-reviewed articles and studies published in English.

### 2.4. Selection Process

The selection process involved two independent reviewers (IA and ARH) who meticulously screened each record and full-text article to determine eligibility based on the inclusion and exclusion criteria. Initially, both reviewers independently assessed the titles and abstracts of the identified studies. Each reviewer then evaluated the full texts of potentially relevant articles to ensure they met the criteria for inclusion. In cases where there was disagreement or uncertainty about a study’s eligibility, the reviewers resolved conflicts by reviewing the articles together. This collaborative approach ensured that all decisions were made with a consensus and adhered to the review’s criteria. Inter-rater reliability was not quantified using a kappa statistic, but the independent review and consensus process ensured consistency in study selection. No automation tools were used in the selection process; instead, the emphasis was on a thorough, manual review to maintain high standards of accuracy and reliability.

### 2.5. Data Extraction and Synthesis

Data were independently extracted by two reviewers (IA and ARH) using a standardized data extraction form developed for this review. Extracted data included study characteristics (e.g., year, country, design), patient characteristics (e.g., stage, subtype), ctDNA detection methods, treatment context, and reported survival outcomes. Discrepancies between reviewers were resolved by consensus through discussion. No automation tools were used in the data extraction process.

#### 2.5.1. Outcomes

Data were collected on key survival outcomes, including recurrence-free survival (RFS), defined as the interval from the designated baseline to the first locoregional or distant recurrence of breast cancer; disease-free survival (DFS), defined as the time from surgery to the first invasive disease recurrence, the diagnosis of a second primary invasive cancer, or death from any cause; invasive disease-free survival (IDFS), defined as the duration from definitive surgery to the first occurrence of an invasive breast cancer event (locoregional, distant, or contralateral) or death, excluding in situ events; distant recurrence free-survival (DRFS), defined as the time interval between the date of patient consent for treatment and the date of clinical diagnosis of metastatic recurrence or death by any cause; and overall survival (OS), defined as the time from randomization or surgery to death from any cause [47]. Effect measures reported in the included studies primarily included hazard ratios (HRs) with 95% confidence intervals (CIs), as well as Kaplan–Meier survival estimates. These were extracted and summarized qualitatively to compare the prognostic implications of ctDNA detection across studies.

We note that recurrence-free survival (RFS), disease-free survival (DFS), invasive disease-free survival (IDFS), and distant recurrence-free survival (DRFS) may have overlapping or differing definitions across studies. These differences, such as inclusion of second primary cancers or variations in follow-up periods, were considered during qualitative synthesis to ensure that survival outcomes were interpreted in the context of each study’s specific definitions.

#### 2.5.2. Other Variables

Additional data collected included treatment details, study design, methods for ctDNA sample collection and analysis, breast cancer subtypes (e.g., HR+, HER2+, TNBC), cancer stage, and the number of patients enrolled and available for ctDNA analysis. Information on stage distribution for HR+/HER2− patients and the breakdown of patients based on ctDNA status within this subgroup was also gathered.

#### 2.5.3. Study Risk of Bias Assessment

Due to the heterogeneity of study designs—including cohort studies, case–control studies, and peer-reviewed conference abstracts—a standardized risk-of-bias tool (e.g., QUIPS, Newcastle–Ottawa) was not applied. Instead, risk of bias was assessed qualitatively by evaluating clarity of study design, population selection, ctDNA measurement methodology, and completeness of outcome reporting. This approach ensured systematic consideration of potential selection, reporting, and methodological biases across all included studies.

#### 2.5.4. Synthesis Methods

A qualitative synthesis method was employed to systematically examine and interpret results from the included observational studies. The studies were organized and analyzed according to their findings on ctDNA detection and survival outcomes in HR+/HER2− breast cancer patients undergoing neoadjuvant and adjuvant treatments. Studies were grouped according to the clinical context of ctDNA assessment: neoadjuvant, adjuvant, or post-treatment surveillance. This grouping allowed for evaluation of ctDNA prognostic value across distinct phases of breast cancer therapy. No data conversions were performed. Where summary statistics (e.g., HRs, CIs) were not available, the studies were narratively described without estimation. Missing data were noted and discussed qualitatively when relevant. Results from individual studies were tabulated to highlight study design, treatment context, ctDNA detection methods, timing, and associated survival outcomes. Tables were used to summarize key characteristics and findings for ease of comparison.

#### 2.5.5. Reporting Bias Assessment

As this review involves a qualitative analysis, statistical techniques for assessing publication bias were not utilized. Instead, a thorough literature search was performed to reduce the potential for reporting bias, ensuring that a wide array of studies meeting the inclusion criteria were considered.

#### 2.5.6. Certainty Assessment

Certainty of the evidence was assessed qualitatively, as the review included only observational studies without randomized controlled trials after the screening process was performed, and therefore, the GRADE system was not utilized. While GRADE or adapted tools can be applied in systematic reviews, formal GRADE assessment was not feasible in this review due to the inclusion of only observational studies with heterogeneous designs and outcomes. Certainty of evidence was instead assessed qualitatively by evaluating the consistency of results across studies, reliability of ctDNA detection methods, and completeness of outcome reporting, ensuring a systematic and transparent evaluation of the evidence.

### 2.6. Protocol and Registration

All methods were conducted systematically and transparently according to PRISMA guidelines. All methods were conducted systematically and transparently according to PRISMA guidelines. The protocol was prospectively registered in the Open Science Framework (OSF) registries: https://doi.org/10.17605/OSF.IO/PB3DF.

## 3. Results

### 3.1. Study Selection

**Figure 1:**
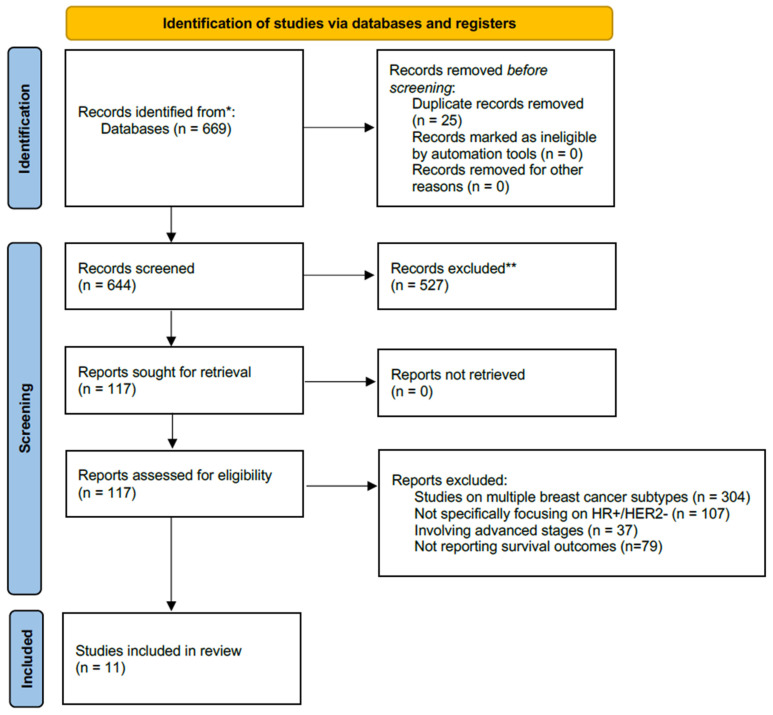
PRISMA 2020 flow diagram for new systematic reviews, which included searches of databases and registers only. * Records identified through database searches in Ovid MEDLINE and Embase (2000–3 May 2024). Reference lists of relevant articles and reviews were also screened. ** Records excluded at title/abstract screening for not meeting eligibility criteria (e.g., wrong population, intervention, comparator, outcome, or study design).

The review process began with 644 articles and abstracts. After initial screening, 117 articles were selected by excluding those not addressing the impact of ctDNA on recurrence/disease/invasive disease-free survival and overall survival, not focusing on stages I–III breast cancer, or not specific to HR+/HER2− breast cancer. A detailed evaluation then further narrowed the list by excluding studies on multiple breast cancer subtypes or those not specifically focusing on HR+/HER2−, as well as those involving advanced stages. This process resulted in 11 articles that met all criteria, focusing exclusively on the impact of ctDNA on survival in HR+/HER2− breast cancer stages I–III (Figure 1).

### 3.2. Reporting Results

The total number of HR+/HER2− breast cancer patients included in the studies was 1656. The stage distribution among these patients was as follows: stage I accounted for 115 patients (6.9%), stage II accounted for 157 patients (9.4%), and stage III accounted for 160 patients (9.6%). The remaining 1224 patients (73.9%) were reported across multiple stages (e.g., stages I–III). The breakdown of tumor grades was grade 1 with 57 patients (3.4%), grade 2 with 129 patients (7.8%), and grade 3 with 114 patients (6.9%). The remaining 1356 patients (81.8%) had unknown grades. Available demographic information, including age and menopausal status, was inconsistently reported across the included studies. Menopausal status was not explicitly documented in any of the reports. Where available, median ages ranged from approximately 34 to 72 years, reflecting the inclusion of both younger and older patients. However, the majority of studies did not specify these details, which limits generalizability. A full summary of study characteristics is presented in Supplementary Table S1.

### 3.3. Neoadjuvant Setting

Four studies involving 287 patients investigated the impact of ctDNA detection in the context of neoadjuvant therapy on survival (Table 1). Magbanua et al. (2023) [48] observed that a high cell-free DNA (cfDNA) concentration after neoadjuvant chemotherapy (taxane and anthracycline/cyclophosphamide (T-AC) regimens) was linked to significantly worse DRFS, with a hazard ratio (HR) of 5.89 (95% CI 2.68–12.98). ctDNA was analyzed in plasma using the Signatera personalized and tumor-informed test, which detects up to sixteen patient-specific somatic mutations selected from whole-exome sequencing from treatment-naïve samples [48]. Similarly, Lin et al. (2021) used a panel of fourteen frequently mutated genes in breast cancer and found that ctDNA positivity after neoadjuvant therapy was associated with significantly inferior RFS in estrogen receptor-positive HER2− breast cancer, though exact statistical significance was not reported [49]. On the other hand, Li et al. (2020) [50] used plasma and tumor samples that underwent deep targeted sequencing of 1021 cancer-related genes using a next-generation sequencing panel. They found no significant difference in DFS or OS based on baseline ctDNA status before neoadjuvant chemotherapy in HR+ patients, reporting hazard ratios of 1.75 (95% CI 0.23–13.39) for DFS and 1.66 (95% CI 0.095–28.94) for OS [50]. In addition, Chen et al. (2021) [51] used a PCR amplification method followed by sequencing to obtain the whole genome length and found that ctDNA positivity (<0.1% vs. ≥0.1%) following neoadjuvant endocrine therapy with exemestane was linked to significantly worse OS, with a hazard ratio of 4.4 (95% CI 1.1–4.3) [51]. Interpretation of these findings is limited by substantial heterogeneity in ctDNA assay platforms and outcome measures, which complicates direct comparisons across studies and precludes meta-analytic synthesis.

### 3.4. Adjuvant Setting

Seven studies involving 1369 patients investigated the impact of ctDNA detection in the context of adjuvant therapy on survival (Table 2). In the PENELOPE-B trial, reported by Turner et al. (2023) [52], patients who received neoadjuvant chemotherapy and had residual disease were randomized to adjuvant treatment with either endocrine therapy plus palbociclib or placebo [52]. The study demonstrated that ctDNA detection at baseline and following adjuvant treatment was strongly associated with lower rates of invasive disease-free survival (iDFS), with an HR of 8.8 (95% CI, 3.3–23.4) at baseline and 25.5 (95% CI, 6.5–99.6) at cycle 7. A tumor sample underwent exome sequencing, and up to 50 tumor-specific somatic mutations were monitored in plasma using error-corrected sequencing alongside a proprietary algorithm for ctDNA detection (RaDaR). Garcia-Murillas et al. (2019) [53] sequenced tumor DNA using a panel targeting 14 known driver breast cancer genes. ctDNA detection following surgery and before adjuvant chemotherapy was highly prognostic for RFS in early-stage breast cancer, but specific hazard ratios were not detailed [53]. Using a personalized panel of plasma droplet digital PCR assays, Fiegl et al. (2005) found that persistence of *RASSF1A* methylation following adjuvant therapy with tamoxifen correlated with poor RFS, with a hazard ratio of 6.9 (95% CI 1.9–25.9) [54]. As a tumor suppressor gene, *RASSF1A* regulates cell cycle and apoptosis, and its silencing through methylation is a common epigenetic alteration that may signal a more aggressive disease and therapy resistance.

In a retrospective analysis by Lipsyc-Sharf et al. (2022) [55], the personalized RaDaR assay found that among patients with high clinical or genomic risk breast cancer who were diagnosed at least five years prior to the study and had no known recurrence at the time of analysis, ctDNA positivity following adjuvant therapy (radiation, endocrine therapy, or chemotherapy) was associated with poorer RFS. Specific hazard ratio values were not provided [55]. Olsson et al. (2015) reported that ctDNA levels detected using a personalized panel that identified patient- and tumor-specific chromosomal rearrangements through whole-genome sequencing were predictive of poor disease-free survival (odds ratio 2.1 per doubling of ctDNA, 95% CI 1.3–∞) and overall survival (odds ratio 1.3 per doubling, 95% CI 1.03–1.9) [56]. In addition, in a study including patients with T1–2 N0 tumors, Kujala et al. (2020) reported that a high cfDNA mutation burden following surgery and radiotherapy treatments was associated with poor RFS, with a hazard ratio of 2.23 (95% CI 1.16–4.27) [57]. Primary tumor sequencing was used to detect somatic mutations from 106 cases associated with metastatic breast cancer, which were then followed using ctDNA. Lastly, in the monarchE trial, Loi et al. (2024) found that among patients who had a ctDNA+ test at any timepoint (before treatment and 3, 6, and 24 months following adjuvant treatment with abemaciclib + ET), 87% had an IDFS event in comparison to 15% with a persistent ctDNA-negative (−) status during the study [58]. ctDNA detection was performed using the personalized, tumor-informed Signatera ctDNA assay.

Overall, these findings underscore the prognostic value of ctDNA in predicting poorer outcomes for HR+/HER2− breast cancer patients, reinforcing its potential as a biomarker for assessing disease progression and treatment response. However, as in the neoadjuvant setting, differences in ctDNA assay methodologies and survival endpoints reduce comparability across studies and limit the ability to perform formal quantitative synthesis.

### 3.5. Risk of Bias Assessment

The risk of bias in the included studies was assessed qualitatively, focusing on common sources of bias in observational designs. Across studies, the most frequent concerns were selection bias (driven by small sample sizes and selective inclusion of high-risk subgroups), measurement bias (stemming from differences in ctDNA assay platforms and thresholds for positivity), and reporting bias (related to incomplete survival data or lack of hazard ratio reporting). Attrition bias was also observed in some studies with limited follow-up or incomplete longitudinal sampling.

Specifically, the study of Li et al. (2020) [50] was limited by a very small cohort (n = 21), raising concerns about selection bias and underpowering of survival analyses [50]. Lin et al. (2021) also included a small patient population (n = 41) and did not provide hazard ratios, which may reflect reporting bias [49]. Chen et al. (2021) [51] had a larger sample but lacked detailed reporting on several patient characteristics, contributing to concerns about incomplete reporting [51]. Magbanua et al. (2023) [48] was strengthened by its personalized assay but still limited by heterogeneity in treatment regimens and relatively short follow-up, which may introduce attrition bias [48].

In the adjuvant setting, Fiegl et al. (2005) [54] used an older methylation-based assay that may not be directly comparable with more contemporary ctDNA technologies, raising measurement bias concerns. Olsson et al. (2015) included only 20 patients, making the results vulnerable to selection bias and random error [56]. Garcia-Murillas et al. (2019) reported strong prognostic associations but did not provide hazard ratios, raising issues of reporting bias [53]. Kujala et al. (2020) provided hazard ratios but had limited follow-up and lacked detailed demographic reporting [57]. Lipsyc-Sharf et al. (2022) [55] retrospectively analyzed patients more than five years from diagnosis, introducing potential survivor bias and limiting generalizability [55]. Turner et al. (2023, PENELOPE-B) [52] and Loi et al. (2024, monarchE) [58] were large randomized controlled trials, but even these faced limitations: Turner et al. [52] did not report long-term follow-up beyond cycle 7, and Loi et al. did not provide hazard ratios despite a large cohort, limiting quantitative synthesis.

Overall, while most studies were judged to have a moderate risk of bias, the nature of the concerns varied by study, and the heterogeneity in study design, assays, and outcome reporting should be considered when interpreting the prognostic implications of ctDNA detection.

## 4. Discussion

### 4.1. Summary of Findings

This systematic review underscores the potential role of ctDNA as a significant biomarker for predicting clinical outcomes in patients with HR+HER2− breast cancer. This analysis highlights a consistent association across reviewed studies between ctDNA detection and poorer survival outcomes, notably in both neoadjuvant and adjuvant settings. These findings support the hypothesis that ctDNA can provide real-time insights into tumor dynamics, enabling more personalized treatment strategies for patients. This aligns with prior research examining the association between ctDNA detection and prognosis in other breast cancer subtypes (e.g., HER2+ and triple-negative breast cancer) patients [59,60,61,62,63], as well as in other cancer types [64,65,66].

In the neoadjuvant setting, the relationship between baseline ctDNA levels and DFS reinforces the need for further investigation into the predictive value of ctDNA for treatment response. For instance, Magbanua et al. (2023) [48] found that higher pretreatment ctDNA concentrations were associated with variables related to tumor aggressiveness, and ctDNA positivity at any point was associated with inferior DRFS. In addition, among patients with residual disease, negative ctDNA pre-surgery was significantly associated with improved survival compared with those who tested positive for ctDNA. However, the variability in findings underscores the complexity of ctDNA dynamics and its interaction with treatment. For example, Li et al. (2020) [50] reported no significant association between baseline ctDNA (before neoadjuvant therapy) status and DFS or overall survival (OS) in HR-positive patients. These discrepancies may stem from differences in study methodologies, such as sample timing, ctDNA quantification techniques, and patient populations. The timing of ctDNA analysis—whether at baseline, post-treatment, or follow-up—can influence results, as ctDNA dynamics change with treatment. Additionally, variations in assay sensitivity and patient characteristics, like tumor subtype or genetic profiles, could contribute to the observed differences. The type of treatment may also affect ctDNA dynamics, with chemotherapy potentially clearing ctDNA differently than endocrine therapy, although this remains uncertain. These factors highlight the need for standardized methods and careful consideration of patient variables in future research [47,67,68,69,70].

In the adjuvant setting, the significant associations observed between ctDNA detection and IDFS further validate the prognostic value of ctDNA. Studies by Turner et al. (2023) [52] and Chen et al. (2021) [51] revealed that ctDNA positivity post-therapy correlated with worse survival outcomes, suggesting that monitoring ctDNA could aid in identifying patients at higher risk of recurrence. However, in the study by Turner, most patients had metastases on staging at the time of ctDNA positivity. Moreover, the persistence of ctDNA post-treatment could potentially inform clinical decision-making regarding the need for more aggressive therapeutic interventions. Despite the promise of ctDNA as a prognostic biomarker, the variability in the specific methodologies used to assess ctDNA complicates the establishment of standardized cutoffs and clinical guidelines.

### 4.2. Clinical Implications

Despite promising findings, ctDNA currently remains a prognostic rather than a predictive biomarker for HR+/HER2− breast cancer. It is essential to distinguish between ctDNA’s prognostic and predictive utility. A prognostic biomarker provides information about the likelihood of disease recurrence or survival outcomes, independent of treatment, whereas a predictive biomarker guides therapy by indicating which patients are likely to benefit from a specific intervention. Currently, ctDNA functions primarily as a prognostic biomarker. Its detection is consistently associated with a higher risk of disease recurrence and poorer survival outcomes in both neoadjuvant and adjuvant settings. However, there is insufficient evidence that modifying treatment based on ctDNA results leads to improved clinical outcomes. Therefore, ctDNA cannot yet be considered a predictive biomarker capable of guiding treatment choices in routine clinical practice. This distinction is vital, and caution is needed before integrating ctDNA into routine practice. The updated ASCO liquid biopsy guidelines, set for release in 2025, are expected to reinforce the role of ctDNA in risk assessment, rather than as a tool for therapeutic intervention, aligning with previous ASCO recommendations for further validation [71,72]. Additionally, the psychological impact of positive ctDNA results—especially in the absence of clinical evidence of disease—can cause significant patient anxiety and uncertainty [73]. To mitigate psychological distress, clinicians should incorporate patient counseling, provide clear explanations of test implications, and engage patients through shared decision-making frameworks. Structured communication strategies—including discussing uncertainties, outlining monitoring plans, and emphasizing patient preferences—can help reduce anxiety associated with biomarker testing. Integrating psychosocial support, such as referrals to oncology social workers or support groups, may further help patients navigate the emotional implications of ctDNA monitoring and improve overall patient-centered care. Also, false positives or detection of minimal residual disease may lead to overdiagnosis and unnecessary interventions. Clear communication about these limitations is essential for ethical patient care.

### 4.3. Limitations

This systematic review has several limitations that should be acknowledged. The first important limitation relates to the relatively small sample sizes of several of the included studies. While the overall pooled cohort comprised 11 studies with 1644 patients, individual study populations were often modest. Such small sample sizes limit statistical power and may increase susceptibility to random variation, thereby restricting the generalizability of the findings. Consequently, the conclusions of this review should be interpreted with caution. Future research efforts should prioritize large, multi-center studies with adequate sample sizes to provide more robust and widely applicable evidence. Another key limitation of this review is the variability in ctDNA assays and testing procedures across the included studies. Differences in assay sensitivity, sequencing techniques, and the timing of sample collection could significantly influence the detection of ctDNA and thus the reported prognostic associations. Some studies used highly sensitive next-generation sequencing platforms, whereas others relied on PCR-based assays, which may have lower sensitivity for detecting low-frequency variants. Additionally, variations in pre-analytical factors such as blood processing, plasma storage, and timing of sample collection relative to surgery or adjuvant therapy could contribute to inconsistent results. This methodological heterogeneity complicates direct comparisons between studies and may limit the generalizability of our findings. Future studies employing standardized ctDNA testing protocols are needed to confirm and extend the prognostic relevance of ctDNA in HR+/HER2− early-stage breast cancer. Furthermore, some studies evaluated multiple breast cancer subtypes. In these cases, it was sometimes difficult to fully isolate the HR+/HER2− population, which resulted in exclusion of potentially relevant studies. Finally, the prognostic role of ctDNA has not yet been validated in large, prospective clinical trials, and its predictive utility in guiding treatment decisions remains uncertain.

### 4.4. Future Directions

Future research should focus on large-scale, multi-center studies to standardize ctDNA testing protocols, including optimal timing, quantification methods, and cutoff thresholds for predicting clinical outcomes. Head-to-head trials comparing different ctDNA assays are needed to identify the most accurate and clinically feasible platforms. Combining ctDNA with other biomarkers or multi-omics approaches could enhance insights into tumor heterogeneity and resistance. Harmonized data reporting and use of common outcome measures would enable individual patient-level meta-analyses and facilitate cross-study comparisons. Additionally, the development of more sensitive and specific ctDNA assays is needed to reduce false positives and improve clinical utility. Clinical trials exploring ctDNA-guided therapy, particularly early intervention based on ctDNA detection, are crucial to assess whether it can improve survival outcomes. Prospective intervention trials where ctDNA detection guides adjuvant therapy escalation or early intervention are particularly important to determine whether ctDNA-guided strategies can improve survival outcomes (Table 3). Notable ongoing studies include **MiRaDoR** (NCT05708235), which evaluates molecular relapse-free survival with ctDNA-guided early intervention; **DARE** (NCT04567420), assessing the efficacy of ctDNA-guided palbociclib plus fulvestrant; and **TBCRC-068** (NCT06923527), testing elacestrant in ctDNA-detected relapse. These trials may provide critical evidence for ctDNA-guided escalation strategies and help refine post-relapse treatment approaches. Addressing the psychological impact of ctDNA results, especially in the absence of clinical disease, will also be vital for ethical patient care. Ultimately, more evidence is needed to establish the predictive value of ctDNA and justify its use in routine clinical decision-making. Current trials are actively investigating this aspect, and until more robust evidence supports its predictive value, ctDNA should remain a prognostic tool, with further research required to establish its clinical utility.

## 5. Conclusions

This systematic review highlights the growing evidence supporting circulating tumor DNA (ctDNA) as a prognostic biomarker in early-stage hormone receptor-positive, HER2-negative breast cancer. Across both neoadjuvant and adjuvant treatment settings, ctDNA detection was generally associated with poorer survival outcomes, although findings were variable across studies due to differences in assay methods, timing, and patient populations. This underscores its potential utility for risk stratification and surveillance while acknowledging that current evidence is insufficient to guide routine clinical practice. Heterogeneity in ctDNA detection methods, timing, and study populations limits broad clinical application at this time. While ctDNA currently serves as a prognostic rather than predictive biomarker, ongoing trials may clarify its role in guiding therapeutic decisions. Standardization of assays, timing, and reporting is essential to realize ctDNA’s full potential in personalized breast cancer management. Ultimately, these findings should inform the design of clinical trials evaluating ctDNA-guided interventions rather than being interpreted as justification for immediate changes in patient care.

## Figures and Tables

**Table 1:** Studies in the neoadjuvant setting.

Study	Number of Patients (n)	ctDNA Methods	Outcome Metrics	Findings	HR (95% CI) If Reported
**Li et al. (2020) [50]**	48	Deep targeted sequencing of 1021 cancer-related genes using a next-generation sequencing panel	DFS and OS	No correlation between ctDNA detection after neoadjuvant chemotherapy and survival outcomes	1.75 (95% CI 0.23–13.39) for DFS and 1.66 (95% CI 0.095–28.94) for OS
**Lin et al. (2021) [49]**	47	Deep targeted sequencing of fourteen frequently mutated genes in breast cancer using a next-generation sequencing panel	RFS	ctDNA detection after neoadjuvant chemotherapy was associated with worse outcomes	No HRs reported
**Chen et al. (2021) [51]**	49	ctDNA was amplified using PCR followed by sequencing to obtain the whole genome length and to determine ctDNA content percentages in plasma. Classification was set as positive or negative (<0.1% or ≥0.1% for ctDNA content)	OS	ctDNA detection after neoadjuvant therapy (endocrine therapy with exemestane) was associated with worse outcomes	4.4 (95% CI 1.1–4.3)
**Magbanua et al. (2023) [48]**	46	ctDNA was detected in plasma using a personalized and tumor-informed test (STAR Methods)	DRFS	ctDNA detection after neoadjuvant chemotherapy was associated with worse outcomes	5.89 (95% CI 2.68–12.98)

**Table 2:** Studies in the adjuvant setting.

Study	Number of Patients (n)	ctDNA Method of Detection	Outcomes	Findings	HR (95% CI) If Reported
Fiegl et al. (2005) [54]	148	Personalized dPCR assays were used for analyzing RASSF1A methylation levels in plasma	RFS	ctDNA detection after adjuvant therapy (tamoxifen) is associated with worse survival outcomes	6.9 (95% CI 1.9–25.9)
Lipsyc-Sharf et al. (2022) [55]	83	Whole-exome sequencing on primary tumors was conducted to identify somatic mutations that could be tracked via ctDNA	RFS	ctDNA detection after adjuvant therapy (radiation, endocrine therapy, or chemotherapy) is associated with worse survival outcomes	No HRs reported
Olsson et al. (2015) [56]	20	A personalized panel for ctDNA level quantification was used	DFS and OS	ctDNA levels after adjuvant therapy are associated with worse survival outcomes	2.1 per doubling of ctDNA (95% CI 1.3–∞) for DFS; 1.3 per doubling of ctDNA (95% CI 1.03–1.9) for OS
Garcia-Murillas et al. (2019) [53]	51	The primary tumor was sequenced to detect somatic mutations, and personalized tumor-specific digital PCR assays were employed to track these mutations in sequential plasma samples	RFS	ctDNA detection during adjuvant therapy (after surgery, before chemotherapy) is associated with worse survival outcomes	No HRs reported
Kujala et al. (2020) [57]	79	Primary tumor sequencing was used to detect somatic mutations, which were then followed in ctDNA	RFS	ctDNA detection after adjuvant therapy (surgery followed by radiotherapy) is associated with worse survival outcomes	2.23 (95% CI 1.16–4.27)
Turner et al. (2023) [52]	78	Exome sequencing of plasma using the personalized RaDaR assay	iDFS	ctDNA detection after adjuvant therapy (endocrine therapy) is associated with worse survival outcomes	25.5 (95% CI 6.5–99.6)
Loi et al. (2024) [58]	910	ctDNA detection was performed using the personalized, tumor-informed Signatera ctDNA assay (Natera, Inc.)	iDFS	Among patients who had a ctDNA+ test at any timepoint (before treatment and 3, 6, and 24 months following adjuvant treatment with abemaciclib + ET), 87% had an IDFS event compared to 15% with persistent ctDNA-negative (−) status	No HRs reported

**Table 3:** Summary of ongoing trials investigating the predictive and prognostic value of ctDNA in early-stage breast cancer.

Trial Name	Population	NCT Number	Phase	Estimated Completion	Primary Outcome	Potential Impact on Evidence Base
MiRaDoR	HR+/HER2− early-stage BC	NCT05708235	II	2028	Molecular relapse-free survival	May provide evidence for ctDNA-guided early intervention
DARE	HR+/HER2− early-stage BC	NCT04567420	II	2027	Efficacy of ctDNA-guided palbociclib + fulvestrant	Could support ctDNA-guided second-line therapy
TBCRC-068	HR+/HER2− early-stage BC	NCT06923527	II	2027	Efficacy of elacestrant in ctDNA-detected relapse	May inform post-relapse treatment strategies

## Data Availability

No new data were generated or analyzed in this study. All data supporting the findings of this review are derived from previously published studies, which are cited throughout the manuscript.

## Supplementary Materials

The following supporting information can be downloaded at https://www.mdpi.com/article/10.3390/cancers17172831/s1. Table S1: Key characteristics of studies reporting circulating tumor DNA (ctDNA) in early-stage HR+/HER2− breast cancer.
