# Supplementary material for: Prognostic Value of Circulating Tumor DNA in HR+/HER2− Stage I–III Breast Cancer: A Systematic Review
Source: Cancers (Basel). 2025 Aug 29;17(17):2831. doi: 10.3390/cancers17172831 (PMC12427406; doi:10.3390/cancers17172831)
Supplement: Supplementary file 1 [file cancers-17-02831-s001.zip › cancers-3771274-supplementary.pdf]

Supplementary

Table S1. Key characteristics of studies reporting circulating tumor DNA (ctDNA) in early-stage HR+/HER2− breast cancer.

Study	Treatment received	Endpoints	Duration of follow-up	Histology (grade)	Breast cancer subtypes included	Stage distribution for ER+/HER2−	Evaluable patients for ctDNA within HR+/HER2−
Magbanua 2024	Neoadjuvant chemotherapy	DRFS, RCB	Median 3.1 years (range 0.46–7.6)	Grade 1/2: 56; Grade 3: 62; Unknown: rest	Multiple	T1/T2: 81; T3/T4: 39; Node-positive: 55; Node-negative: 61; Unknown: rest	T1/T2: High cfDNA 39, Low cfDNA 42; T3/T4: High 19, Low 20
Turner 2023	Palbociclib + endocrine therapy	iDFS, DMFS	Median 42.9 months	NS	HR+/HER2−	NS	Baseline ctDNA+: 7; Cycle 7 ctDNA+: 4; detailed kinetics reported
Li 2020	Neoadjuvant chemotherapy	DFS, OS	Median 46 months (range 11–68)	NS	Multiple	NS	ctDNA+: 10; ctDNA−: 19
Lin 2021	Neoadjuvant chemotherapy	RFS	Median 5.1 years	NS	Multiple	Stage II: NS; Stage III: NS	ctDNA+: 11; ctDNA−: 30
Fiegl 2005	Adjuvant therapy (tamoxifen)	RFS, OS	Median 3.6 years (range 0.2–9.7)	Grade I: 47; II: 83; III: 14; Unknown: 4	HR+/HER2−	Grade-based distribution: Grade I: 47; II: 83; III: 14; Unknown: 4	ctDNA+: 29; ctDNA−: 119
Lipsyc-Sharf 2022	Adjuvant therapy (surgery/radiation/endocrine)	RFS	Median 10.4 years	Grade 1: 11; 2: 42; 3: 30	HR+/HER2−	IIA: 2; IIB: 24; IIIA: 40; IIIB: 3; IIIC: 14	ctDNA+: 8; ctDNA−: 75
Chen 2021	Adjuvant therapy (endocrine with exemestane)	OS	Up to 24 months or death	NS	HR+/HER2−	Stage II: NS; Stage III: NS	ctDNA+: 32; ctDNA−: 48
Garcia-Murillas 2019	Neoadjuvant chemotherapy ± surgery	RFS	Median 35.5 months (IQR 27.9–43.0)	NS	Multiple	NS	NS
Olsson 2015	Adjuvant therapy	DFS, OS	Median 9.2 years	Grade 1: 1; 2: 11; 3: 8	HR+/HER2−	Stages I–III	NS
Kujala 2020	Adjuvant radiotherapy post-surgery	NS	NS	Grade 1: 17; 2: 48; 3: 14	HR+/HER2−	Stage I: 60; II: 19	NS
